# Genetic landscape of a neonatal hypogonadotropic hypogonadism series: Novel variants and phenotypic spectrum

**DOI:** 10.1111/jne.70274

**Published:** 2026-09-24

**Authors:** Karine Aouchiche, Laure Van Wormhoudt, Kevin Perge, Joanne Dornberger, Cindy Colson, Marine Delagrange, Clara Leroy, Jennifer Ledmazel, Vanessa Menut, Jessica Benarrous Jaillet, Djamel Bendifallah, Mohamad Hage Chehade, Christelle Dib, Anne Barlier, Thierry Brue, Rachel Reynaud, Alexandru Saveanu

**Affiliations:** ^1^ Assistance Publique‐Hôpitaux de Marseille (APHM), Department of Paediatrics, Paediatric Endocrinology Unit CHU Timone Enfants Marseille France; ^2^ Multidisciplinary Pediatric Department Aix Marseille Univ, APHM, INSERM, MMG, UMR 1251, La Timone Children's Hospital Marseille France; ^3^ Hospices Civils de Lyon (HCL), Pediatric endocrinology Unit, Hôpital Femme Mère Enfant Université Claude Bernard Lyon 1 Lyon France; ^4^ Department of Pediatric Endocrinology, Genetic, and Gynecology University Hospital of Toulouse Children's Hospital Toulouse France; ^5^ Department of Genetics University Hospital of Lille Lille France; ^6^ Department of Pediatrics Endocrinology and Diabetology University Hospital of Bordeaux Bordeaux France; ^7^ Department of Pediatric Endocrinology, Diabetology Metabolism University Hospital of Lille Lille France; ^8^ Department of Pediatrics Annecy Genevois Hospital Epagny Metz‐Tessy France; ^9^ Department of Pediatrics Endocrinology and Diabetology University Hospital of Nantes Nantes France; ^10^ Hospices Civils de Lyon (HCL), Pediatric Genetic Unit, Hôpital Femme Mère Enfant Université Claude Bernard Lyon 1 Lyon France France; ^11^ Department of Pediatrics Alpes Leman Hospital Center Contamine‐sur‐Arve France; ^12^ Department of Pediatrics Boulogne sur Mer Hospital Center Boulogne sur Mer France; ^13^ Department of Pediatrics Elbeuf‐Louviers‐Val de Reuil Intercommunal Hospital Center Normandy France; ^14^ Laboratory of Molecular Biology GEnOPé Aix Marseille Univ, APHM, INSERM, MMG, UMR 1251, La Timone University Hospital Marseille France; ^15^ Department of Endocrinology Aix Marseille Univ, APHM, INSERM, MMG, MarMaRa Institute, UMR 1251, La Conception University Hospital Marseille France

**Keywords:** gene panel analysis, genetic variants, Kallman syndrome, congenital hypogonadotropic hypogonadism

## Abstract

To describe clinical presentation and genetic findings in a cohort of infants with congenital hypogonadotropic hypogonadism (CHH) diagnosed before 2 years of age. From a large cohort of patients who underwent next‐generation sequencing (NGS) for CHH between 2019 and 2025, we identified all patients tested at ≤2 years of age. Genetic analysis consisted of a targeted CHH gene panel, followed by a CPHD‐NGS panel. In one remaining patient with neurodevelopmental defects, whole‐exome sequencing was performed. Phenotypic data were collected through the GENHYPOPIT registry. Twelve male infants (1.5% of the total cohort) met the inclusion criteria; one additional patient via familial screening was included, for a total of 13 patients. All had micropenis; four had associated cryptorchidism (bilateral in half of cases). CHH was biologically confirmed during mini‐puberty in 10 infants, at a median age of 6.8 weeks. Seven of these 10 infants received gonadotropin therapy, at an average of 4.1 months. MRI revealed bilateral olfactory bulb agenesis in five cases. A pathogenic or likely pathogenic variant was identified in 7 of 12 patients (58%), involving *TACR3* (*n* = 2), *ANOS1*, *FGFR1* (including two novel variants), *SOX10*, *GLI2* (through the CPHD panel), and *SOX11* (identified by exome sequencing). A genetic cause was identified in 58% of cases, a diagnostic yield higher than reported in pubertal cohorts. Early genetic diagnosis allowed anticipatory monitoring for syndromic manifestations and associated pituitary deficiencies—particularly relevant for *FGFR1* and *GLI2* variant carriers at risk of CPHD. Our findings support the inclusion of *GLI2* and *SOX11* in targeted HH gene panels.

## 1 INTRODUCTION

Congenital hypogonadotropic hypogonadism (CHH) is an extremely rare condition with an estimated incidence between 1/15,000 and 1/50,000 individuals, predominantly males.^1^ CHH is caused by impaired secretion of the two principal gonadotropins, luteinizing hormone (LH) and follicle‐stimulating hormone (FSH),^2^ due to deficient production, secretion, or action of gonadotropin releasing hormone (GnRH), a hypothalamic peptide that regulates their secretion.^3^ In boys, CHH can manifest in the neonatal period as micropenis and/or cryptorchidism,^4^ and subsequently affects pubertal development and future fertility.^5^ In newborn boys therefore, observation of micropenis and/or unilateral or bilateral cryptorchidism should lead to hormonal assessment during mini‐puberty (between 1 and 4 months of age) to diagnose CHH.^6^ Early diagnosis is important to initiate gonadotropin treatment, which has been shown to promote the development and descent of the testicles and should thus improve future fertility.^7^ However, limited data mean that there is currently no consensus on treatment protocols.^8^ Unfortunately, most cases of CHH are not diagnosed in the first year of life, but later, in adolescence or early adulthood,^3^, ^9^, ^10^ when pubertal delay or infertility become apparent, leading to significant psychosocial and reproductive sequelae.^4^, ^5^

CHH can be challenging to diagnose,^11^ but genetic analyses can contribute alongside clinical and laboratory findings.^3^ Since the 1990s, several genes have been implicated in CHH: *KAL1*/*ANOS1*, associated with Kallmann syndrome (KS) (i.e., CHH with hypo/anosmia),^3^, ^12^, ^13^ *GNRHR*, associated with normosmic CHH^9^, ^14^ and *FGFR1*, associated with both KS and normosmic CHH, highlighting the phenotypic spectrum of the disease.^15^, ^16^, ^17^ More recently, the development of next‐generation sequencing (NGS) techniques has led to a wider set of associations, with up to 40 genes potentially involved.^1^, ^3^, ^9^, ^10^ Moreover, genes involved in combined pituitary deficiency (CPHD), for instance *PROP1* and *GLI2*, have been shown to also be associated with CHH, either in isolation, or as the first sign of future combined deficiency, supporting the concept of a developmental and phenotypic continuum between CHH and CPHD.^18^, ^19^, ^20^, ^21^

Identifying a genetic cause allows for more accurate prognosis and genetic counselling,^1^, ^3^ and this is currently achieved in about 20 to 40% of cases of CHH.^3^, ^11^, ^17^, ^22^ However, data on genetic diagnosis in infancy are limited, with most series focusing on older patients.^3^, ^4^, ^10^ We therefore investigated the yield of genetic testing in very young CHH patients and describe the corresponding genotype–phenotype correlations.

## 2 METHODS

### 2.1 Study design

A retrospective analysis was conducted of phenotypic and genetic data in the GENHYPOPIT database of patients who underwent genetic testing for hypogonadism hypogonadotropic (HH) between May 2019 and January 2025 at age ≤2 years. GENHYPOPIT is an international network established to collect phenotypic data and perform genetic analyses on patients with congenital hypopituitarism including those with CHH.^23^ All patients were from 10 French hospitals participating in GENHYPOPIT. All participants provided written consent for genetic testing. This study is in accordance with the declaration of Helsinki. Data were anonymously collected in a database declared to health authorities in accordance with local regulations. Declaration was made to the National Commission for Data Protection and Liberties (CNIL‐France): 1991429v0. data.

### 2.2 Genetic analyses

All index cases were analyzed by NGS with a panel of genes associated with HH: *ANOS1 (KAL1)* (NM_000216), *AXL* (NM_021913), *CHD7* (NM_017780), *FEZF1* (NM_001024613), *FGF8* (NM_033163), *FGF17* (NM_003867), *FGFR1* (NM_023110), *GNRH1* (NM_000825), *GnRHR* (NM_000406), *HS6ST1* (NM_004807), *IGSF10* (NM_178822), *IL17RD* (NM_017563), *KISS1* (*NM_002256*), *KISS1R* (NM_032551), *NSMF* (NM_015537), *PROK2* (NM_001126128), *PROKR2* (NM_144773), *SEMA3A* (NM_006080), *SOX2* (NM_003106), *SOX10* (NM_006941), *TAC3* (NM_013251), *TACR3* (NM_001059), and *WDR11* (NM_018117).

If no pathogenic (P) or likely pathogenic (LP) variant was identified in these tests, patients underwent a second round of genetic testing using the NGS panel of genes linked to CPHD, notably *GLI2* (NM_005270), *PROP1* (NM_006261) (full listing of genes available in Supplementary Data S1).

Exons and 20‐nucleotide intronic terminals were amplified using a QIAseq targeted DNA custom panel library (Qiagen, Hilden, Germany) according to manufacturer recommendations. Samples were sequenced using a NextSeq1000 instrument (Illumina, San Diego, CA, USA) with the P1‐SBS‐300 cycles reagent kit (Illumina). Alignment and variant calling were performed with the CLC Genomic Workbench software (Qiagen) and SEQUENCE Pilot (JSI medical systems GmbH). All variants were annotated in the GRCh37 human genome with HGVSc and HGVSp nomenclatures according to international recommendations. Variants were classified as pathogenic (P), likely pathogenic (LP), of uncertain significance (VUS), likely benign (LB), or benign (B), according to American College of Medical Genetics (ACMG) guidelines^24^ (Supplementary Data S2). Allele frequencies were obtained from GnomAD (genome aggregation database) version 4.1.0. Public databases of clinically relevant variants were also consulted (HGMD version 2023.4, ClinVar and LOVD). In silico pathogenicity analysis predictions were combined using the aggregator program MobiDetails.^25^

Nonsense‐mediated mRNA decay (NMD) was assessed using NDMEscPredictor (https://nmdprediction.shinyapps.io/nmdescpredictor/). Functional impact was assessed using in vitro reports from the literature, if available. P and LP SNVs in index cases and relatives were confirmed by Sanger sequencing, using primers specific to the relevant exons on an ABI 3500xl DxGenetic Analyzer (Thermo Fisher Scientific, primers available on request).

### 2.3 Patient phenotypes

Phenotypic data were obtained from detailed questionnaires completed by physicians on inclusion of patients in the GENHYPOPIT database. The clinical data collected included anthropometric parameters at birth, abnormal clinical findings, and family and personal medical history. Laboratory data from assessments of anterior pituitary axes during mini‐puberty, or later if the patient was referred to an endocrinologist at a later stage, were also studied. The patient was considered small for gestational age (SGA) if birth weight was below the 10th percentile for gestational age^26^ and large for gestational age (LGA) if their birth weight was above the 90th percentile for gestational age.^27^ Micropenis was defined as penile length ≤25 mm at term.^28^ HH diagnoses were established or suspected by referring pediatric endocrinologists on observation of micropenis and/or cryptorchidism, and typically confirmed by hormonal assessment during mini‐puberty. HH was defined as testosterone < 2 nmol/L with decreased or normal serum gonadotropin (LH and FSH) levels during mini‐puberty (between 4 weeks and 6 months of age).^3^ Age‐specific reference ranges during male mini‐puberty were obtained from Rohayem et al. (2024), with values relevant to the age range of interest provided in Supplementary Data S3.^29^, ^30^ Anti‐Müllerian hormone (AMH, pmol/L) and inhibin B (pg/mL) concentrations were collected when available. In cases of late referral to an endocrinologist, after mini‐puberty, genetic testing was performed based on clinically suspected HH. In accordance with European guidelines, the etiological workup for HH included abdominal ultrasound and cerebral magnetic resonance imaging (MRI).^3^ The cerebral MRI findings considered included the size of the pituitary gland, presence of extra‐pituitary cerebral malformations (optic nerve, olfactory bulbs or corpus callosum abnormalities), pituitary stalk interruption syndrome, and ectopic posterior pituitary.^31^

### 2.4 Statistical analysis

Continuous variables were expressed as median with interquartile range (IQR). Categorical variables were summarized as numbers and percentages.

## 3 RESULTS

Between May 2019 and January 2025, 774 patients underwent targeted NGS of genes involved in CHH. Among them, 12 patients (1.5%) were 2 years old or younger at the time of genetic testing, all males. The median age at genetic testing among these 12 patients was 8.1 months (IQR 6.2–19.3 months).

### 3.1 Genetic findings (Figure 1)

**FIGURE 1:**
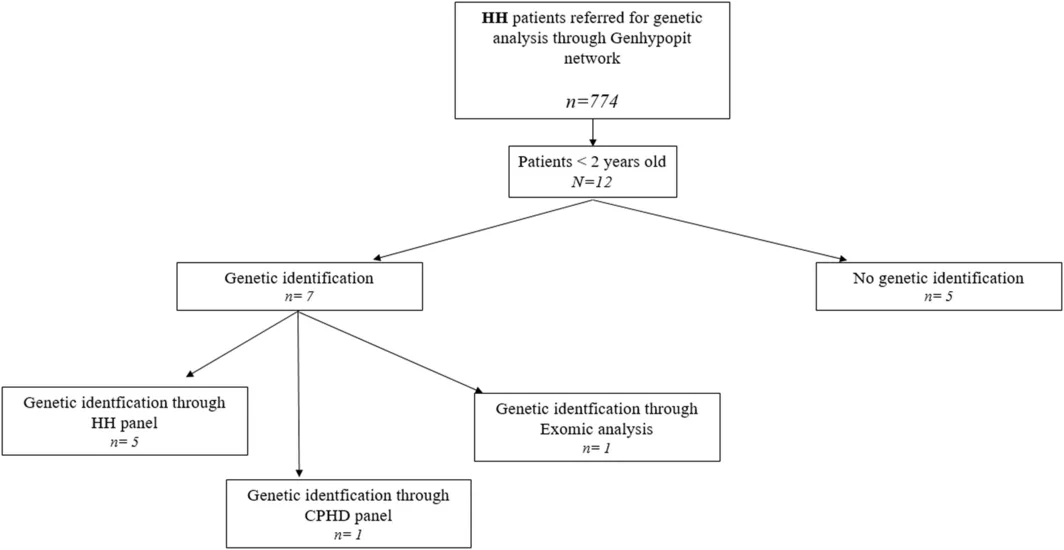
Genetic findings in our infant cohort of HH patients.

A genetic cause for CHH (P or LP variant) was identified in 7/12 index cases (58%, Table 1). In five cases, the genetic cause was identified using CHH panel screening: *TACR3* variants in two cases (patient 1A and 2), and *ANOS1*, *FGFR1*, *SOX10* variants in one case each. In one case (patient 6), the causal variant, a LP variant in *GLI2*, was identified at the second step, using the CPHD NGS panel. In one case finally (patient 7), a *SOX11* variant was identified by whole exome analysis for neurodevelopmental disorder and then confirmed by Sanger sequencing (Supplementary Data S4). Six further VUS were identified in four genes (Supplementary Data S5).

**TABLE 1:** Pathogenic and likely pathogenic variants in our cohort.

Patient	Gene	Exon	c.	p.	ACMG classification	ACMG classification criteria^a^	MAF (Popmax)	Statut	Transmission
1A	*TACR3* (NM_001059) (NP_001050.1)	Exon 3	c.824G>A	p.(Trp275*)	P	PVS1 PM2 PP5	0.06 (Eur)	Homozygous	Autosomal recessive^b^
1B		Exon 3	c.824G>A	p.(Trp275*)	P	PVS1 PM2 PP5	0.06 (Eur)	Homozygous	Autosomal recessive
2		Exon 1	c.324G>T	p.(Trp108*)	P	PVS1 PM2 PM3 PP5	0	Heterozygous	Autosomal recessive
		Exon 3	c.824G>A	p.(Trp275*)	P	PVS1 PM2 PM3	0.06 (Eur)	Heterozygous	Autosomal recessive
3	*ANOS 1* (NM_000216) (NP_000207.2)	Exon 8	c.1065del	p.(Gln356Lysfs*4)	P	PVS1 PM2 PP4	0	Hemizygous	Not tested^c^
4	*FGFR1* (NM_023110) (NP_075598.2)	Exon 3	c.289G>A	p.(Gly97Ser)	LP	PS3 PM2 PM1 PP3 PP5	0	Heterozygous	De novo
5	*SOX10* (NM_006941) (NP_008872.1)	Exon2	c.326A>C	p.(Asn109Thr)	LP	PM1 PM2 PM5 PP3	0	Heterozygous	Not tested
6	*GLI2* (NM_005270) (NP_005261.2)	Exon 13	c.2614_2615dup	p.(Ser873Alafs*40)	LP	PVS1 PM2	0	Heterozygous	Autosomal dominant, maternally inherited
7	*SOX11* ^d^ (NM_003108.4) (NP_003099.1)		c.191G>C	p.(Arg64Pro)	P	PM1, PM2, PM5, PP3_Strong, PP5_Very_Strong	0	Heterozygous	De Novo

*Note*: Minor allele frequency (MAF) is expressed in %.

^a^ ACMG criteria listed in Supplementary Data S1.

^b^ Non‐consanguineous parents.

^c^ Consanguineous parents, maternal uncle, and cousin with Kallmann syndrome and synkinesia.

^d^ Initially identified by exome sequencing for neurodevelopmental disorder and confirmed by Sanger sequencing in Marseille.

**FIGURE 2:**
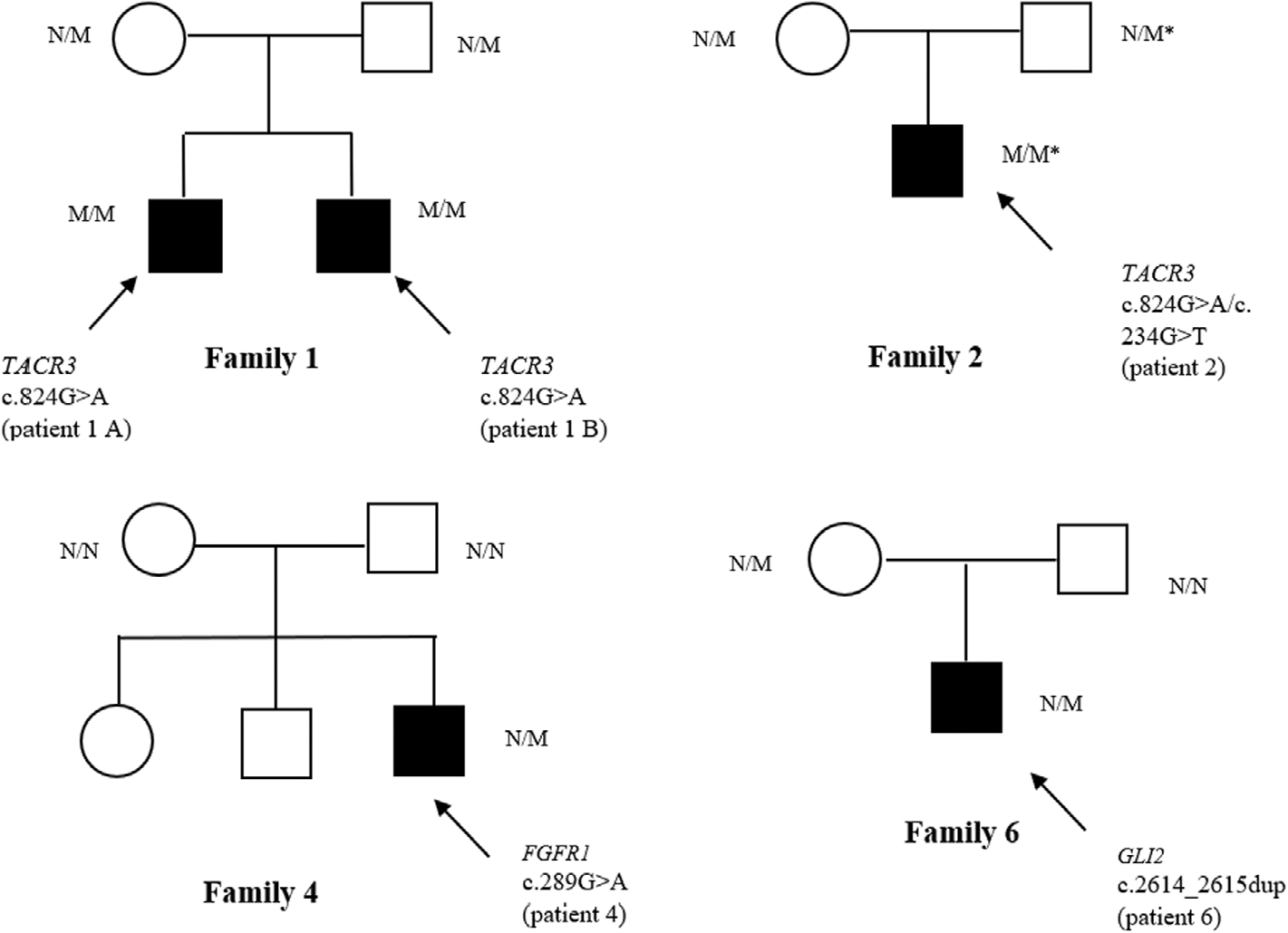
Family pedigrees showing segregation of LP or P variants identified in CHH children. Squares, male family members; circles, females; filled black symbols, affected subjects. Genotypes: M/M, homozygous; M/M*, compound heterozygous; N/M or N/M*, heterozygous; N/N, normal.

Family segregation analysis was performed in nine relatives from four families (index case 1A, 24 and 6 (Figure 2)). In family 1, the proband's brother (patient 1B) underwent targeted analysis of the *TACR3* gene, which identified the same homozygous *TACR3* P variant (Table 1), inherited from their heterozygous parents (non consanguineous). Patient 4's parents were tested negative for patient 4's *FGFR1* variant, suggesting de novo occurrence. Patient 6's *GLI2* LP variant was found to be inherited from his apparently healthy mother.

Family history of hypogonadism was identified (without family testing) in two patients (2/12 in total, 16.7%): patient 3, with a LP variant in *ANOS1*, has a maternal uncle and a cousin with KS and synkinesis; patient 8, in whom no P or LP variant was identified, was conceived through assisted reproductive technology and his mother has a history of hypogonadism and required gonadotropin injections to initiate pregnancy.

### 3.2 Phenotype characteristics (Table 2 and Figure 3 *)*

**TABLE 2:** Phenotypical characteristics of patients.

Patient	Genetic cause diagnosed	Micropenis	Cryptorchidism	Midline anomaly	Other associated conditions	Age at hormonal evaluation (week)	Testosterone (nmol/L)	LH/FSH (UI/L)	Cerebral MRI	Age at first genetic analysis (week)	Age at treatment initiation (week)	Treatment (duration) Surgical management of cryptorchidism (if applying)
1A	*TACR3 P variant*	Yes	No	No	None	6	<0.45	0.07/08	Normal	13	13	Gonadotropins (4 months)
1B	*TACR3 P variant*	Yes	Yes, Unilateral	No	None	7	0.21	0.09/1.1	Not performed	8	17	Gonadotropins (4 months) Surgery
2	*TACR3 P variant*	Yes	No	No	SGA^a^	56	1.32	Not performed	Normal	122	/	None
3	*ANOS 1 P variant*	Yes	Yes Bilateral	No	None	7	0.49	<0.3/<0.3	Not performed	27	27	Testosterone Surgery
4	*FGFR1 LP variant*	Yes	No	No	Macrocephaly, NDD^a^	8	0.56	<0.3/<0.3	Anterior pituitary hypoplasia	57	22	Testosterone
5	*SOX10 LP variant*	Yes	Yes Unilateral	No	Sd de Waardenburg type 2	7	1.01	<0.3/1.2	Artefacted	110	6	Gonadotropins (4 months) Surgery
6	*GLI2 LP variant*	Yes	No	No	None	7	0.35	1.6/0.2	Bilateral olfactory bulb agenesis	133	19	Gonadotropins (5 months)
7	*SOX11 P variant*	Yes	No	Yes^b^	Pierre Robin sequence, NDD	7	0.39	<0.3/<0.2	Unilateral olfactory bulb agenesis	12	17	Gonadotropins (5 months)
8	No genetic identification	Yes	No	No	Antenatal right multicystic renal dysplasia	33	<0.45	<0.05/<0.2	Not performed	32	/	None
9	No genetic identification	Yes	No	No	LGA^a^	8	<0.10	<0.3/<0.3	Olfactory bulb hypoplasia	24	18	Testosterone
10	No genetic identification	Yes	Yes bilateral	No	None	36	0.28	<0.3/<0.3	Olfactory bulb agenesis	69	/	None Surgery
11	No genetic identification	Yes	No	No	None	6	<1/39	0.2/0.7	Olfactory bulb agenesis	26	21	Gonadotropins (6 months)
12	No genetic identification	Yes	No	No	Trigonocephaly	5	0.76	0.17/1	Not performed	32	16	Gonadotropins (6 months)

^a^ NDD, neurodevelopmental disorder; SGA, small for gestational age; LGA, large for gestational age.

^b^ Atrial septal defect.

**FIGURE 3:**
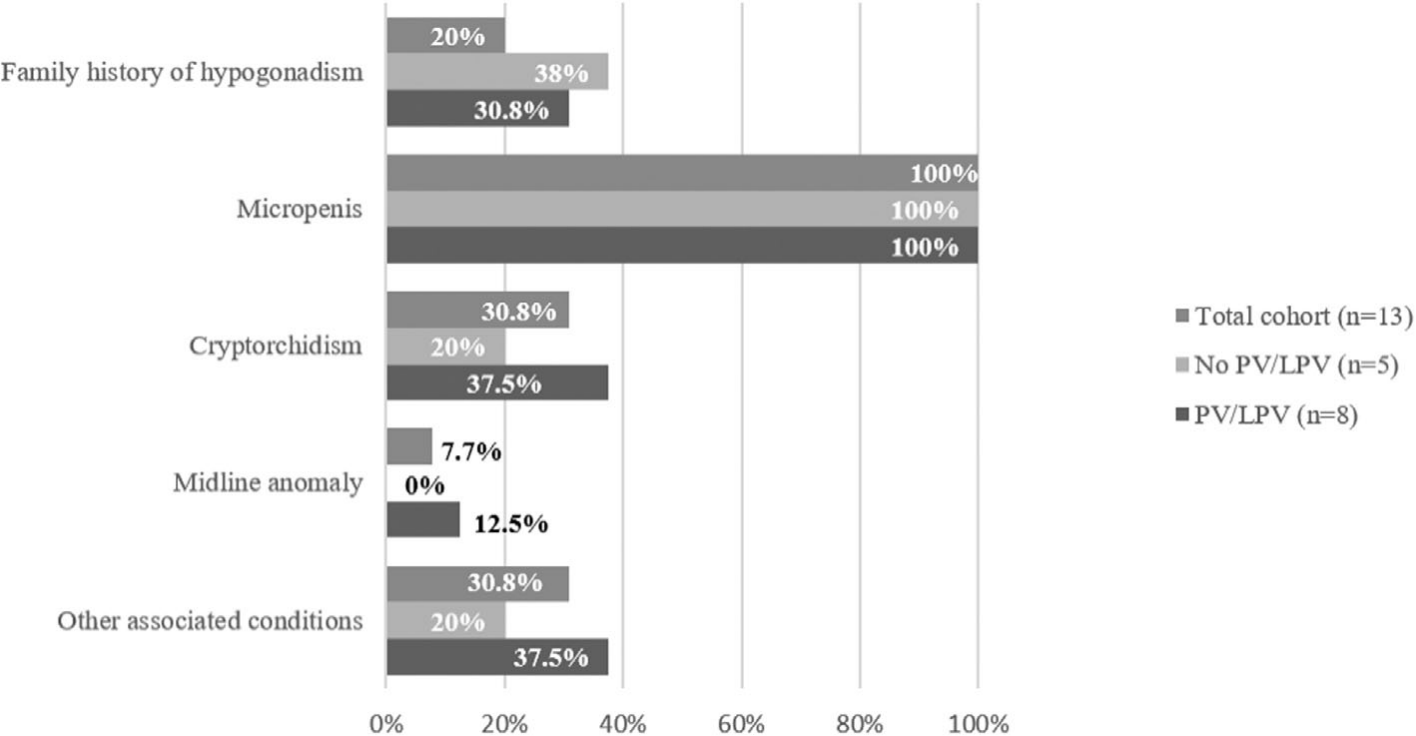
Phenotypic characteristics of the CHH cohort.

Patient 1A's brother, patient 1B, was included in the phenotypic analysis because he was assessed before 2 years of age. All infants were born at term. Eleven out of 13 infants (85%) were appropriate for gestational age, one was SGA, and one was LGA. Twelve out of 13 patients were conceived naturally.

All 13 patients presented with micropenis in the neonatal period. Cryptorchidism was observed in four patients (31%), bilaterally in two cases and unilaterally in the other two.

CHH diagnosis was confirmed by laboratory tests during mini‐puberty in 10/13 patients (77%). The median age at the first laboratory assessment was 6.8 weeks (IQR, 6.4–7.6 weeks). Median serum testosterone was 0.52 nmol/L (IQR, 0.37–0.95 nmol/L), with low FSH (median, 0.5 IU/L; IQR, 0.3–1.0 IU/L) and low LH levels (median, 0.2 IU/L; IQR, 0.1–0.3 IU/L). AMH concentrations, were low or low‐normal for age (median, 274 pmol/L; IQR, 126–426. pmol/L). Inhibin B levels were also low for age (median, 81.5 pg/mL; IQR, 57.5–104 pg/mL).

In the remaining three patients (23%), the median age at clinical suspicion of HH was 9.2 months (IQR, 8.0–13.0 months), too old for biological mini‐puberty exploration of CHH.

Laboratory findings are summarized in Supplementary Data S6. No thyrotropic, somatotropic, or corticotropic deficiencies were identified in the study group at the time of genetic analysis.

Pituitary MRI was performed in 9/13 patients (69%) (Table 2). Five patients (5/9, 56%) had unilateral or bilateral olfactory bulb agenesis, among whom just two had an identified genetic cause.

Renal ultrasonography was performed in 11/13 patients (85%) and revealed renal agenesis in one patient (9%), patient 8, who had no identified P or LP variant.

Two patients had syndromic HH. Patient 5 had Waardenburg‐like features and patient 7 had CHH, neurodevelopmental impairment, craniofacial anomalies, and congenital heart disease. Other notable clinical features were autism spectrum disorder, language delay, and macrocephaly in patient 4, and antenatally diagnosed multicystic renal dysplasia in patient 8.

### 3.3 Genotype–phenotype correlations

All three patients with *TACR3* variants had truncating variants, homozygous in patient 1A and 1B and compound heterozygous in patient 2. They had no symptoms other than those associated with HH. Patient 3 with a truncating *ANOS1* variant also had purely HH genital phenotype; however, the olfactory status of this patient remains undetermined; his age precluded formal testing, and no MRI of the olfactory structures was performed. Patient 4 with a missense *FGFR1* variant had a more complex phenotype, including neurodevelopmental disorder and macrocephaly; additional investigations revealed anterior pituitary hypoplasia without other hormonal deficiencies. Patient 5 with a LP *SOX10* variant had iris hypopigmentation and congenital deafness in addition to micropenis and cryptorchidism, consistent with a diagnosis of Waardenburg syndrome type 2. The only clinical symptom of patient 6, who has a LP *GLI2* variant, was micropenis. However, cerebral MRI revealed bilateral agenesis of the olfactory bulbs. Patient 7, with a *SOX11* variant, had Pierre Robin sequence, atrial septal defect, exotropia, neurodevelopmental disorder, feeding difficulties, and unilateral agenesis of the olfactory bulb.

### 3.4 Treatment

Among the 10 patients with laboratory confirmed CHH, seven (70%) received gonadotropin therapy, initiated at a median age of 4.1 months (IQR, 3.1–4.4 months), including two with cryptorchidism. Among them, four (57%) had a P or LP variant.

Three patients (3/10, 30%) received testosterone supplementation including one with bilateral cryptorchidism, initiated at a median age of 5.2 months (IQR, 4.4–6.2 months). Two of these patients (2/3, 67%) had a P or LP variant.

None of the three patients whose CHH was diagnosed after mini‐puberty received hormonal treatment.

All four patients with cryptorchidism underwent orchiopexy, performed at a median age of 18.5 months (IQR, 15.0–19.5 months).

## 4 DISCUSSION

This study is one of few, to our knowledge, to specifically focus on HH genetic testing in infancy. Among this subgroup of 12 index patients aged ≤2 years identified from our large CHH cohort, the positivity rate of genetic tests by panel was 50% (58% if the *SOX11* variant identified by exome sequencing is included), higher than in most previous studies in adults. In fact, the reported diagnostic yields for both targeted gene panel approaches^32^, ^33^, ^34^, ^35^ and whole‐exome sequencing^36^, ^37^, ^38^ in congenital HH range from 20 to 40%. The higher genetic diagnostic yield in our cohort compared to previous reports may be explained by our inclusion criteria. Indeed, micropenis was a key indication for genetic testing in our study, a clinical feature previously shown to be strongly associated with positive genetic findings in adolescent cohorts.^39^ Furthermore, focusing on infancy avoids the inclusion of patients investigated for HH with a partial phenotype overlapping with constitutional delay of puberty.^40^ The reported positivity rate of another recent French study was even higher, at 75%,^41^ but the classification of the variants was not reported, making study comparison difficult.

Surprisingly, *TACR3* was the only gene mutated in more than one patient in our cohort, despite being rarely reported in large HH series.^42^ *TACR3* encodes neurokinin‐3 receptor (NK3R), which plays a key role in the regulation of kisspeptin release and GnRH secretion.^43^, ^44^ Loss‐of‐function variants in *TACR3* impair this signaling pathway, resulting in hypogonadotropic hypogonadism.^43^ All three patients, in two independent families, carried the same *TACR3* pathogenic variant c.824G>A, p.(Trp275*), the most frequent in ClinVar (ID 66084) likely reflecting a founder effect in the Caucasian population about 2000 years ago.^42^, ^45^, ^46^, ^47^, ^48^ In those patients with HH, variant was either in the homozygous or compound heterozygous state, consistent with autosomal recessive inheritance.^49^ Truncating variants such as this one lead to haploinsufficiency and HH phenotype without additional clinical features, consistent with our patients' clinical presentation.^42^ Patient 2 also had a novel truncating *TACR3* variant, c.324G>A, p.(Trp108*), in trans with p.(Trp275*), and predicted to act by similar mechanism of haploinsufficiency.

We also identified a novel hemizygous variant, c.1065del, in *ANOS1*, a gene encoding anosmin‐1 and associated with the X‐linked form of KS.^12^ The variant involves a single base pair deletion that induces a frameshift resulting in a premature stop codon, predicted to lead to production of a truncated protein, p.(Gln356Lysfs*4), or more likely, to premature mRNA degradation through NMD. *ANOS1* loss‐of‐function variants have been widely described and account for approximately 5%–10% of cases of KS.^50^ The patient reported here had apparently isolated HH; however, genetic analysis was performed at just 6 months of age, too young for investigations of anosmia or imitation synkinesia. Several family members had more typical KS, with HH, anosmia and imitation synkinesia, consistent with the known clinical spectrum associated with *ANOS1* variants and helping for the clinical follow up of the patient.

Loss‐of‐function variants in *FGFR1* are identified in approximately 10%–12% of patients with CHH,^22^, ^51^ making *FGFR1* one of the most frequently implicated genes in this condition.^50^, ^52^ The missense variant, c.289G>A, p.(Gly97Ser), identified in patient 4, has been reported previously^53^, ^54^ and functional studies support its pathogenicity by haploinsufficiency.^54^ Pathogenic variants in *FGFR1* are known to have a wide phenotypic spectrum, ranging from pure HH to KS and other syndromic presentations. In these patients, HH may be complete or partial and even temporary.^16^, ^50^, ^55^ In our patient, HH was clinically isolated at the time of evaluation, at 7 months of age. Interestingly, MRI showed pituitary hypoplasia, as observed previously in patients with *FGFR1‐associated* CPHD.^56^, ^57^, ^58^, ^59^ Indeed, patients with FGFR1 variants may present with midline brain anomalies, including pituitary defects and absence of septum pellucidum.^58^, ^59^ The identification of a pathogenic *FGFR1* variant, given the risk of progression to CPHD, illustrates how early genetic diagnosis directly informs clinical management. In this study, CPHD‐related genes were also investigated in the context of isolated HH for two reasons. First, HH may be an initial manifestation of CPHD, with additional pituitary hormone deficiencies developing over time.^21^ Second, some CPHD genes, such as *GLI2* ^18^ have also been implicated in HH, as highlighted by our report^19^ on the patient with a *GLI2* variant also included in the present study. The risk of progression to CPHD—whether identified through established HH genes such as *FGFR1* or through CPHD‐related genes such as *GLI2*—thus provides a compelling argument for systematic genetic testing in HH presenting in infancy.

One patient underwent whole‐exome sequencing for neurodevelopmental disorder, after negative HH Panel, which identified a *SOX11* variant, confirming that HH can be a feature of a broader syndrome and that more exhaustive genetic analyses are justified in such cases.^36^ Recent data further support the notion that *SOX11* testing should be considered in patients with HH, notably those with intellectual disability^60^ or Coffin–Siris‐like phenotypes involving developmental delay and skeletal and facial defects.^61^ Our patient had Pierre Robin sequence, as already reported in *Sox11*‐null mice.^62^ He also had an atrial defect, a cardiac malformation that, to our knowledge, has never previously been associated with *SOX11* variation. Early identification of pathogenic variants in patients with HH may allow earlier recognition of associated syndromic conditions, enabling anticipatory follow‐up and timely management of emerging disabilities.^60^, ^61^, ^63^, ^64^ Our findings also support prioritizing *SOX10* analysis in patients with HH and hearing impairment.^63^

Compared to the only published neonatal cohort,^41^ genes such as *CHD7*, *KISS1R*, and *GnRHR*, recurrently implicated in CHH cohorts, were not identified in our patients. Given the small size of both cohorts, these differences should be interpreted with caution and may reflect sampling variability rather than true neonatal‐specific genetic distribution.

Our study further supports the inclusion of *GLI2* and *SOX11* in targeted HH gene panels, as these genes are not yet analyzed systematically.^39^

Our study also highlights how rare genetic testing in the neonatal period is, with only 1.5% of patients investigated at 2 years of age or younger in our IHH cohort. This is surprising given the current accessibility of sequencing technologies. The low proportion of young children with HH in the GENHYPOPIT database raises questions regarding pediatricians' training in the detection of clinical signs of HH, particularly in maternity wards. This is especially important because all patients diagnosed during mini‐puberty in our study were able to access either gonadotropin or testosterone treatment.^65^ Several small studies have found that gonadotropins successfully mimic male mini‐puberty, with short‐term evidence of improved testicular function, including Sertoli cell proliferation and promotion of testicular descent.^5^ Indeed, adequate gonadotropin replacement during mini‐puberty may promote testicular descent in infants with CHH and cryptorchidism, potentially avoiding or facilitating orchidopexy by increasing testicular size and mobility.^7^ In cases where full descent is not achieved, as in our study, patients required surgery consistent with international guidelines recommending early intervention for undescended testes between the ages of 6 and 18 months.^66^, ^67^, ^68^, ^69^ Furthermore, patients with lower testicular volumes, lower inhibin B concentrations, and unresolved cryptorchidism tend to respond less favorably to adult fertility treatment.^7^, ^70^ Early diagnosis, particularly combined with systematic genetic testing, may therefore improve patient management with a potentially correlated response to treatment and genotype.

Beyond diagnosis, genotype data may also inform therapeutic strategies. Indeed, the underlying genetic etiology could help predict the natural history of hypogonadism, including the likelihood of spontaneous pubertal development or hormonal reversibility observed in a subset of CHH patients.

Appropriate genetic counselling in CHH families after identification of pathogenic variants is also essential given the marked genetic heterogeneity of the condition, the diversity of inheritance patterns, and the variable penetrance and expressivity of variants.^9^, ^71^

Several limitations of this study should be acknowledged. First, the small sample size limited the generalizability of our findings. Second, longitudinal data on pubertal progression and the potential emergence of additional pituitary deficiencies over time were not systematically available, which does not allow conclusions on long‐term hormonal evolution. Third, our genetic approach relied on successive targeted panels, which, while appropriate for clinical practice, offers more limited diagnostic coverage than whole‐exome or whole‐genome sequencing. As genomic sequencing becomes increasingly accessible, its integration into the diagnostic of neonatal HH may improve diagnostic yield.

## 5 CONCLUSION

This study is among the first to characterize the genetic spectrum of CHH specifically in infancy, a population largely underrepresented in the literature. Our findings highlight the importance of early recognition of micropenis, allowing for timely hormonal evaluation during mini‐puberty and therefore early diagnostic confirmation and treatment. The genetic spectrum identified here in infancy largely overlaps with those reported in adolescent cohorts, with a diagnostic yield of 58%—higher than generally reported in pubertal cohorts; however, early genetic diagnosis enables tailored follow‐up, anticipation of associated pituitary deficiencies and possible syndromic manifestations while also providing appropriate genetic counselling for families. Our findings support the inclusion of *GLI2* and *SOX11* variant screening in HH panel.

## AUTHOR CONTRIBUTIONS

**Karine Aouchiche:** Conceptualization; investigation; writing – original draft; methodology; validation; writing – review and editing; formal analysis. **Laure Van Wormhoudt:** Writing – review and editing; investigation; validation; writing – original draft; methodology; formal analysis. **Kevin Perge:** Resources; writing – review and editing. **Joanne Dornberger:** Writing – review and editing; resources. **Cindy Colson:** Resources; writing – review and editing. **Marine Delagrange:** Writing – review and editing; resources. **Clara Leroy:** Resources; writing – review and editing. **Jennifer Ledmazel:** Resources; writing – review and editing. **Vanessa Menut:** Writing – review and editing; resources. **Jessica Benarrous Jaillet:** Resources; writing – review and editing. **Djamel Bendifallah:** Writing – review and editing; resources. **Mohamad Hage Chehade:** Writing – review and editing; resources. **Christelle Dib:** Resources; writing – review and editing. **Anne Barlier:** Formal analysis; software; writing – review and editing; supervision. **Thierry Brue:** Funding acquisition; supervision; writing – review and editing; conceptualization; project administration. **Rachel Reynaud:** Writing – review and editing; conceptualization; investigation; methodology; validation; supervision. **Alexandru Saveanu:** Conceptualization; investigation; writing – review and editing; visualization; validation; methodology; supervision; formal analysis; software.

## FUNDING INFORMATION

No funding was received for this study.

## CONFLICT OF INTEREST STATEMENT

The authors have no relevant financial or non‐financial interests to disclose.

## ETHICS STATEMENT

This study is in accordance with the declaration of Helsinki. All participants provided written consent for genetic testing. Declaration was made to the National Commission for Data Protection and Liberties (CNIL‐France): 1991429v0.

## Supporting information


**Supplementary Data S1**: Genes analyse by CPHD panel: *ARNT2* (NM_014862), *FGF8* (NM_033163), *FGFR1* (NM_023110), *GH1* (NM_000515), *GLI2* (NM_005270), *HESX1* (NM_003865), *IGSF1* (NM_001555), *LHX3* (NM_014564), *LHX4* (NM_033343), *OTX2* (NM_021728), *PAX6* (NM_000280), *POU1F1* (NM_000306), *PROKR2* (NM_144773), *PROP1* (NM_006261), *SOX2* (NM_003106), *SOX3* (NM_005634), and *TBX19* (NM_005149).
**Supplementary Data S2**. CMG/AMP criteria for variant classification.
**Supplementary Data S3**. References range for AMH and Inhibin B during male mini‐puberty, adapted from Rohayem et al., Endocrine Reviews, 2024.
**Supplementary Data S4**: *SOX11*: c.191G>C sanger sequencing.
**Supplementary Data S5**. Variants of unknown significance identified.
**Supplementary Data S6**. Detailed hormonal characteristics during mini puberty for 10 patients.

## Data Availability

The data that support the findings of this study are available from the corresponding author upon reasonable request.
