# Supplementary material for: Genetic landscape of a neonatal hypogonadotropic hypogonadism series: Novel variants and phenotypic spectrum
Source: J Neuroendocrinol. 2026 Sep 24;38(10):e70274. doi: 10.1111/jne.70274 (PMC13612720; doi:10.1111/jne.70274)
Supplement: Supplementary file 1 [file JNE-38-e70274-s001.docx]

**Supplementary Data**

**Supplementary Data S1** : Genes analyse by CPHD panel: *ARNT2* (NM_014862), *FGF8* (NM_033163), *FGFR1* (NM_023110), *GH1* (NM_000515), *GLI2* (NM_005270), *HESX1* (NM_003865), *IGSF1* (NM_001555), *LHX3* (NM_014564), *LHX4* (NM_033343), *OTX2* (NM_021728), *PAX6* (NM_000280), *POU1F1* (NM_000306), *PROKR2* (NM_144773), *PROP1* (NM_006261), *SOX2* (NM_003106), *SOX3* (NM_005634), and *TBX19* (NM_005149)**.**

**Supplementary Data S2**. CMG/AMP criteria for variant classification

Term Description Evidence of impact (ACMG adapted for disease):

**BS1** Allele frequency greater than expected for disorder

**BS3** Well-established functional studies show no damaging effect

**BS4** Lack of segregation in affected family members

**BP2** In cis with a pathogenic variant in any inheritance pattern Supporting

**BP4** Computational evidence suggesting no impact on gene or gene product

**BP5** Found in a case with alternate molecular basis for disease

**PVS1** Null variant (nonsense, frameshift, canonical +/−1 or 2 splice sites, initiation codon, single or multi-exon deletion) in a gene where loss of function (LOF) is a known mechanism of disease

**PS3** Well-established functional studies supportive of damaging effect Strong

**PM1** Located in a mutational hot spot and/or critical functional domain Moderate

**PM2** Absent from controls (or extremely low frequency)

**PM5** Variant modifying the same aminoacid reported as pathogenic

**PM6** Assumed de novo

**PP1** Segregation in affected family memebers

**PP3** Computational evidence supporting a deleterious effect Supporting

**PP4** Patient’s phenotype is highly specific

**PP5** Reputable source repport as pathogenic

**Supplementary Data S3**. References range for AMH and Inhibin B during male mini-puberty, adapted from Rohayem et al., Endocrine Reviews, 2024

| Parameter | Age | Median/Mean | P 2.5-P97,5 | Sample size | Citation |
| --- | --- | --- | --- | --- | --- |
| Inhibin B, pg/ml  AMH, pmol/L | 0-12 months  29-364 d | 238 (median)  663 (median) | 99-439  288-1242 | N/A  66 | Kelsey et al. – 2016  Jopling et al. - 2018 |

**Supplementary Data S4**: *SOX11*: c.191G>C sanger sequencing

**Supplementary Data S5**. Variants of unknown significance identified

| Patient | Gene | Exon | c. | p. | ACMG classification | ACMG classification criteria* | Statut | Transmission |
| --- | --- | --- | --- | --- | --- | --- | --- | --- |
| 1A | *SEMA3A*  (NM_006080) | Exon 11 | c.1302_1303inv | p.Val435Ile  (NP_006071.1) | VUS | PS3, BS1, BS2 | Heterozygote | Father (unaffected) |
| 7 |  |  |  |  |  |  |  | Not tested |
| 6 |  |  | c.1198 A>G | p.Ile400Val  (NP_006071.1) | VUS | PS3, BS1, BS2 | Heterozygote | Not tested |
| 1A | *WDR11*  (NM_018117) | Exon 10 | c.1303G>A | p.Ala435Thr  (NP_060587.8) | VUS | PM5 | Heterozygote | Mother (unaffected) |
| 8 | *TACR3*  (NM_001059) | Exon 4 | c.974A>G | p.Tyr325Cys  (NP_001050.1) | VUS | PM2 | Heterozygote | Not tested |
| 7 | *SOX3*  (NM_005634) | Exon 1 | c.791T>G | p.Val264Gly  (NP_005625.2) | VUS | PM2 | Hemizygote | Not tested |

*ACMG criteria listed in Supplemental Table 1

Variants classified as VUS were not considerated causative of the CHH phenotype. When available, parental segregation analysis is reported ; « unaffected » refers to absence of clinical features of hypogondotropic hypogonadism.

**Supplementary Data S6**. Detailed hormonal characteristics during mini puberty for 10 patients

| **Variable**  **median [Q1-Q3]** | **Total cohort (n=10)** | **PV/LPV (n=7)** | **No PV/LPV (n=3)** |
| --- | --- | --- | --- |
| **FSH (UI/L)** | 0,5 [0,3 – 1] | 0,3 [0,3-1,1] | 0,7 [0,3-1,0] |
| **LH (UI/L)** | 0,2 [0,1-0,3] | 0,2 [0,09-0,8] | 0,2 [0,2-0,3] |
| **Testosterone (nmol/l)** | 0,53 [0,35-1,32] | 0,49 [0,35-1,35] | 0,76 [0,10-1,39] |
| **AMH (pmol/l) *** | 274,2 [126,4 – 426,3] | 247,2 [194,2-374,1] | 268,5 [58,5-478,4] |
| **Inhibin B (pg/ml) **** | 81,5 [57,5-104,5] | 81,5 [65 – 102] | 110 [50 – 170] |

*Nine concentrations available (missing data for one patient in the PV/LPV group).

** Eight concentrations available (missing data for one patient in the PV/LPV group; inhibin B not measured for one other patient in the PL/LPV group).
